# KCa3.1 inhibition switches the phenotype of glioma-infiltrating microglia/macrophages

**DOI:** 10.1038/cddis.2016.73

**Published:** 2016-04-07

**Authors:** A Grimaldi, G D'Alessandro, M T Golia, E M Grössinger, S Di Angelantonio, D Ragozzino, A Santoro, V Esposito, H Wulff, M Catalano, C Limatola

**Affiliations:** 1 Department of Physiology and Pharmacology, Sapienza University of Rome, Piazzale Aldo Moro 5, Rome 00185, Italy; 2 IRCCS Neuromed, Via Atinense 18, Pozzilli 86077, Italy; 3 Department of Pharmacology, University of California, 451 Health Sciences Drive, GBSF3502, Davis, CA 95616, USA; 4 Center for Life Nanoscience Istituto Italiano di Tecnologia@Sapienza, Rome, Italy; 5 Department of Neurology and Psychiatry, Sapienza University of Rome, Piazzale Aldo Moro 5, Rome 00185, Italy; 6 Pasteur Institute-Department of Physiology and Pharmacology, Sapienza University of Rome, Piazzale Aldo Moro 5, Rome 00185, Italy

## Abstract

Among the strategies adopted by glioma to successfully invade the brain parenchyma is turning the infiltrating microglia/macrophages (M/MΦ) into allies, by shifting them toward an anti-inflammatory, pro-tumor phenotype. Both glioma and infiltrating M/MΦ cells express the Ca^2+^-activated K^+^ channel (KCa3.1), and the inhibition of KCa3.1 activity on glioma cells reduces tumor infiltration in the healthy brain parenchyma. We wondered whether KCa3.1 inhibition could prevent the acquisition of a pro-tumor phenotype by M/MΦ cells, thus contributing to reduce glioma development. With this aim, we studied microglia cultured in glioma-conditioned medium or treated with IL-4, as well as M/MΦ cells acutely isolated from glioma-bearing mice and from human glioma biopsies. Under these different conditions, M/MΦ were always polarized toward an anti-inflammatory state, and preventing KCa3.1 activation by 1-[(2-Chlorophenyl)diphenylmethyl]-1*H*-pyrazole (TRAM-34), we observed a switch toward a pro-inflammatory, antitumor phenotype. We identified FAK and PI3K/AKT as the molecular mechanisms involved in this phenotype switch, activated in sequence after KCa3.1. Anti-inflammatory M/MΦ have higher expression levels of KCa3.1 mRNA (*kcnn4*) that are reduced by KCa3.1 inhibition. In line with these findings, TRAM-34 treatment, *in vivo*, significantly reduced the size of tumors in glioma-bearing mice. Our data indicate that KCa3.1 channels are involved in the inhibitory effects exerted by the glioma microenvironment on infiltrating M/MΦ, suggesting a possible role as therapeutic targets in glioma.

Among primary brain tumors, glioblastoma multiforme (GBM) is the most aggressive form. Even after surgical ablation and routine treatments with chemo- and radio-therapeutic agents, patient survival remains <15 months.^1^ A wide spectrum of new therapeutic strategies is under investigation, including genetic and immunological approaches, but the need for new possible targets remains urgent. As in other tumors, the immune system has a central role in GBM, contributing to tumor progression and metastasis;^2^ for this reason, a novel research field is focused on immunotherapy-based drug discovery. Microglia represent important constituents of the innate immune system of the nervous system. However, in GBM, this first-line defensive system is hijacked by cytokines released by the tumor.^3^

Under physiological conditions, microglia continuously monitor the surrounding parenchyma with active movement of cell processes that extend and retract to sense potentially toxic local alterations.^4, 5^ In addition to this patrolling phenotype, mainly associated with a ramified shape, microglia assume additional intermediate states, culminating in a fully amoeboid shape.^6^ The transition from ramified to amoeboid shape, together with the acquisition of specific functional and molecular characteristics, generates several intermediate phenotypes that represent different activation states: the extremes of this transition comprise, in analogy with the dichotomy described for macrophages, dynamic changes from a pro-inflammatory, M1-like phenotype, to an anti- inflammatory one, namely M2, including a plethora of sub-phenotypes (M2a, M2b, M2c).^7^

Tumor-associated microglia/macrophages (M/MΦ) are polarized toward an anti-inflammatory phenotype under the action of cytokines and factors released by tumor cells^8, 9, 10^ and contribute to further release of soluble factors, which influence tumor growth and behavior.^11, 12^ A wide range of studies aim to induce tumor- associated macrophages to switch from the pro-tumor to the antitumor pro-inflammatory phenotype.^13^

One promising target to control microglia phenotype is the Ca^2+^-activated K^+^ channel (KCa3.1), which is involved in microglia activation in different pathological conditions such as glioma,^14^ spinal cord injury,^15^ ischemia^16^ and Alzheimer's Disease.^17^ In particular, KCa3.1 blockade reduced infarct area and glioma invasiveness in rodent models of cerebral ischemia and cerebral tumor^14, 16^ and reduced the neurotoxicity of microglia upon lipopolysaccharide (LPS) or oligomeric amyloid *β* exposure.^18, 19^ Interestingly, KCa3.1 expression level negatively correlates with patient prognosis.^20^

In this paper, we investigated the effect of KCa3.1 inhibition on the phenotype switching of microglia induced by GBM-conditioned medium (GCM) or by interleukin (IL)-4, a cytokine involved in microglia polarization toward the anti-inflammatory phenotype. We demonstrated that, upon KCa3.1 inhibition by 1-[(2-Chlorophenyl)diphenylmethyl]-1H-pyrazole (TRAM-34), the GBM- or IL-4-induced profile of M/MΦ is more polarized toward an inflammatory phenotype. This effect is confirmed, *ex vivo*, in human specimens from glioma patients and, *in vivo*, in a mouse model of glioma, where TRAM-34 treatment induced an increase of pro-inflammatory and a reduction of anti-inflammatory gene expression in infiltrating CD11b^+^ cells. This polarization corresponded to a reduced tumor size in TRAM-34-treated glioma-bearing mice, further supporting the antitumor effect of inflammatory M/MΦ cells. We also described the molecular mechanisms involved in these modulatory effects, demonstrating the involvement of the focal adhesion kinase (FAK) and phosphoinositide-3 kinase/protein kinase B (PI3K/AKT) signaling pathways.

## Results

We have previously demonstrated that KCa3.1 expression on glioma cells is involved in tumor cell migration in cerebral parenchyma.^14^ As KCa3.1 channels in the brain are also expressed by microglia and infiltrating macrophages (M/MΦ), we now investigated the effect of KCa3.1 blockage on M/MΦ cells, with particular interest on the acquisition of pro-tumor or antitumor phenotype.

**Figure 1:**
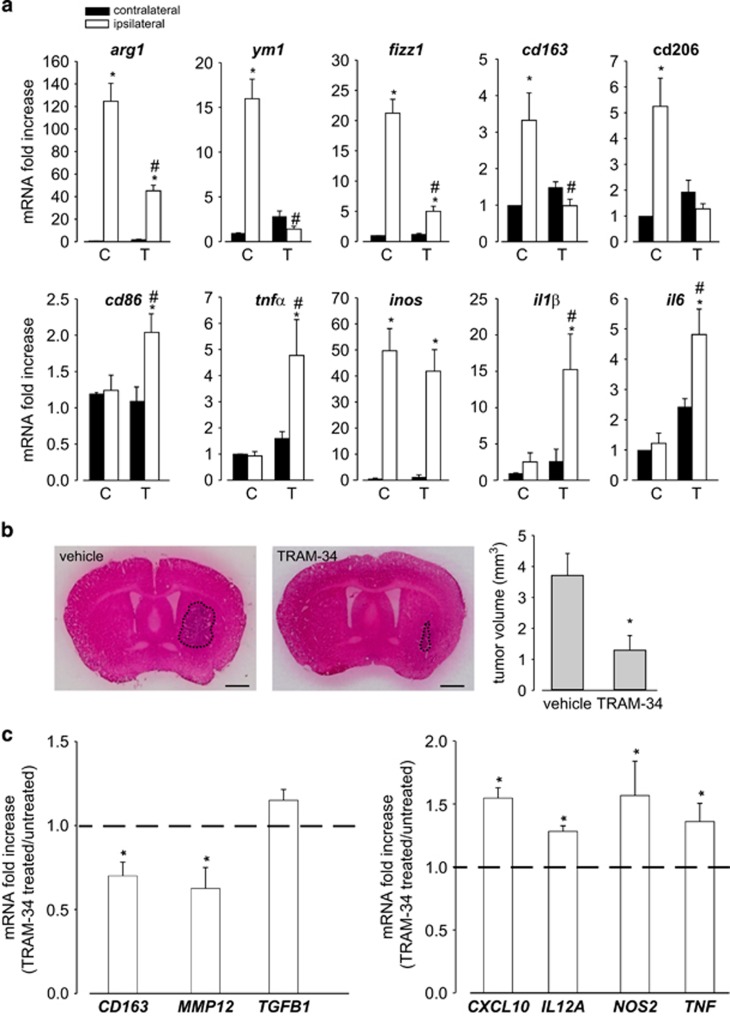
KCa3.1 inhibition modifies the polarization of CD11b^+^ cells extracted from glioma-bearing mice and from biopsies of human GBM. (**a**) RT-PCR of M2-like (*arg1*, *ym1*, *fizz1*, *cd163* and *cd206*) and M1-like (*cd86*, *tnfα*, *inos*, *il1β* and *il6*) related genes in CD11b^+^cells extracted from ipsilateral and contralateral cerebral hemispheres of GL261-bearing mice treated with vehicle (C) or TRAM-34 (T, 120 mg/kg/die). Data are the mean±S.E.M., *P<0.05 *versus* C contralateral; ^#^*P*<0.05 *versus* C ipsilateral by two-way ANOVA; *N*=8. (**b**) Tumor volumes in the brain of mice treated with vehicle or TRAM-34. Data are the mean±S.E.M., *P<0.05 by Student's *t*-test; *N*=8. Scale bar: 1 mm. (**c**) RT-PCR for human M2-like (*CD163*, *MMP12*, *TGFB1*) and M1-like (*CXCL10*, *IL12A*, *NOS2*, *TNF*) related genes expressed by CD11b+ cells extracted from human GBM specimens and treated for 24 h with TRAM-34 (2.5 *μ*M). Data are expressed as fold change of TRAM-34-treated *versus* untreated samples and are the mean±S.E.M., *P<0.05 by Student's *t*-test; *N*=4

It is known that the M/MΦ cell population is deeply influenced by soluble factors produced by tumor cells.^9, 10^ Data in Figure 1a indicate that CD11b+ cells (mainly represented by M/MΦ) isolated from the brain hemisphere ipsilateral to glioma cells injection do express typical anti-inflammatory genes (*arg1*, *ym1, fizz1*, *cd163* and *cd206*), with pro-tumor activity that, upon TRAM-34 treatment, undergo a significant reduction of expression. Interestingly, pro-inflammatory, anti-tumor genes (*cd86*, *tnfα*, *inos*, *il1β* and *il6*), whose expression is not different in the two cerebral hemispheres, regardless of tumor presence (with the only exception being represented by *inos*), are remarkably increased in the glioma ipsilateral hemisphere of TRAM-34-treated mice. Interestingly, when TRAM-34-treated mice were analyzed for tumor growth, a significant reduction of tumor volume was observed (Figure 1b).

Similar results were obtained when CD11b+ cells were isolated from tumoral tissue obtained from GBM patients undergoing surgery: upon TRAM-34 treatment, these cells had a significant reduction of human anti-inflammatory markers (CD163, MMP12) and a significant increase of human pro-inflammatory markers (CXCL10, IL12A, NOS2 and TNF)^21^ (Figure 1c), confirming in human GBM the data obtained in glioma-bearing mice.

**Figure 2:**
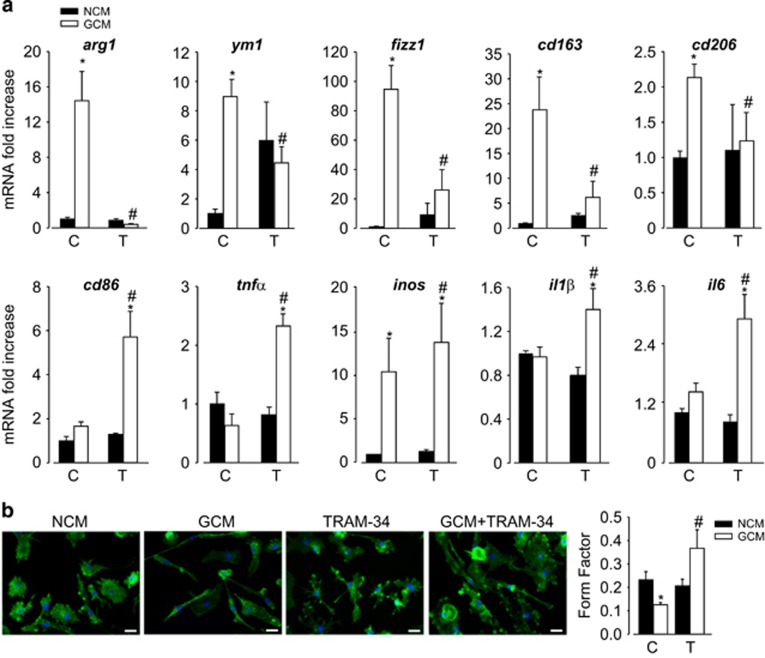
KCa3.1 inhibition reverts GCM-induced microglia polarization. (**a**) RT-PCR on mRNAs of microglia treated with NCM or GCM in the absence (C) or presence of TRAM-34 (T, 2.5 *μ*M), analyzed for the expression of M2-like (*arg1*, *ym1*, *fizz1*, *cd163* and *cd206*) and M1-like (*cd86*, *tnfα*, *inos*, *il1β* and *il6*) related genes. Data are expressed as fold increase and are the mean±S.E.M.; *P<0.05 *versus* NCM; ^#^*P*<0.05 *versus* GCM/C by two-way ANOVA and paired *t*-test for *inos*; *N*=8. (**b**) NCM- and GCM-treated microglia in the absence (C) or presence of TRAM-34 (T, 2.5 *μ*M), stained with phalloidin (green) and Hoechst (blue). Scale bar: 20 *μ*m. Form factor values (calculated as reported in the Materials and Methods section) are shown in the graph and are the mean±S.E.M., *P<0.05 *versus* NCM; ^#^*P*<0.05 *versus* GCM/C by two-way ANOVA; *N*=4

To better investigate the molecular mechanisms involved in this potassium channel-dependent phenotype switch of M/MΦ, we used primary murine microglia exposed to GCM or non-conditioned medium (NCM) for 24 h. Data shown in Figure 2a show that, similarly to what observed *ex vivo*, cell exposure to GCM induced an increased expression of anti-inflammatory genes; TRAM-34 treatment reduced their expression and increased the transcription of pro-inflammatory genes. Similar phenotypic switch was observed when cultured microglia were polarized by cytokine treatment (48 h with LPS/interferon-*γ* (IFN*γ*) or IL-4) and treated with TRAM-34; also, in these conditions the expression of anti-inflammatory genes was reduced with an increase of pro-inflammatory genes upon KCa3.1 inhibition (Supplementary Figure S1A).

The activation state of microglia has been often correlated with their shape, although it is not possible to strictly associate a morphology to a specific phenotype.^6^ A ramified microglia has often been related to a less active, resting phenotype, in comparison with round, amoeboid, active cells. However, this simplified view has been challenged by the description of the continuous monitoring activity of ramified microglia, demonstrating that these cells are not really resting and should probably rather be called 'surveillant'.^4^ We measured the ramification grade of microglia calculating the 'form factor', a parameter taken as 1 for round cells and correspondingly <1 when the morphology deviates from the spherical shape. Figure 2b shows that microglia exposed to GCM have a decreased form factor *versus* NCM-exposed cells and that TRAM-34 treatment significantly reverts this effect. Similar results were obtained when microglia were treated *in vitro* with LPS/IFN*γ* or IL-4 (Supplementary Figure S1B).

**Figure 3:**
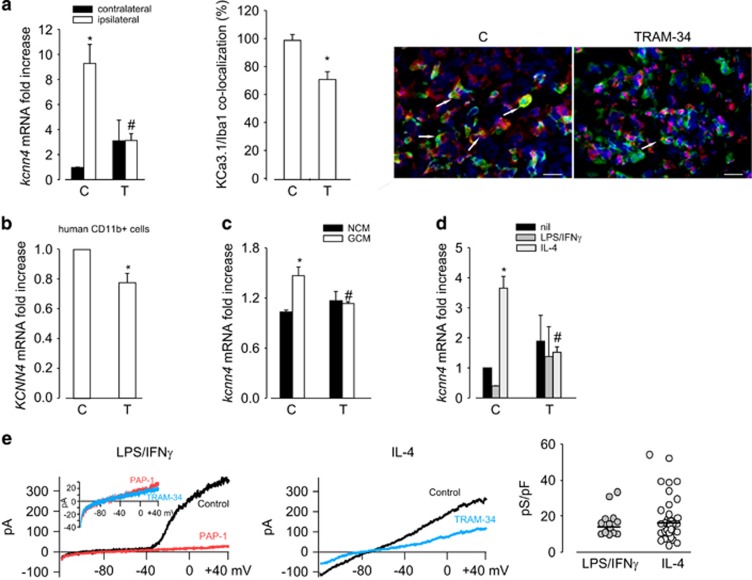
*kcnn4* expression in microglia and infiltrating M/MΦ is correlated with different activation states. (**a**, left) RT-PCR of *kcnn4* expression in CD11b+ cells extracted from the ipsilateral and contralateral cerebral hemispheres of glioma-bearing mice treated with vehicle (C) or TRAM-34 (T, 120 mg/Kg/die). Data are the mean±S.E.M., *P<0.05 *versus* ipsilateral, ^#^*P*<0.05 *versus* contralateral/C by two-way ANOVA; *N*=8. (**a**, center) KCa3.1 expression in Iba1^+^ cells in the tumor area of glioma-bearing mice treated with vehicle (C) or TRAM-34 (T, 120 mg/Kg/die). Data are the average fluorescence intensity of merged signals±S.E.M. and are expressed as the percentage of C, *P<0.05 by Student's *t*-test; *N*=*4*. Representative images are shown on the right (KCa3.1 in red, Iba1 in green), with the arrows indicating some merged signals; scale bar=20 *μ*m. (**b**) RT-PCR of *KCNN4* expression in CD11b^+^ cells extracted from human GBM specimens untreated (C) or treated with TRAM-34 (T, 2.5 *μ*M) for 24 h. Data are expressed as fold change of TRAM-34-treated *versus* untreated samples and are the mean±S.E.M. *P<0.05 by Student's *t*-test; *N*=4. (**c**) RT-PCR of *kcnn4* expression in microglia exposed to NCM and GCM or (**d**) treated with LPS/IFN*γ* and IL-4 in the absence (C) or presence of TRAM-34 (T, 2.5 *μ*M); *P<0.05 *versus* NCM, #*P*<0.05 *versus* GCM/C by two-way ANOVA in (**c**); *P<0.05 *versus* nil, #*P*<0.05 *versus* LPS/IFN*γ*/C or *versus* IL4/C by two-way ANOVA in (**d**); data are expressed as fold increase and are the mean±S.E.M.; *N*=4. (**e**) Typical current traces in response to repeated voltage ramps from −120 to +40 mV (holding potential −80 mV). Each panel shows superimposed currents from microglia treated with LPS/IFN*γ* and IL-4 before (black) and after TRAM-34 (1 *μ*M, blue) perfusion. The prominent Kv current in LPS/IFN*γ*-treated microglia was first blocked with 1 *μ*M PAP-1. On the right, scattered plot representing TRAM-34-sensitive current density in LPS/IFN*γ* (*N*=15) and IL-4 (*N*=35) treated microglia

To test whether microglia phenotypes correlate with different expression levels of KCa3.1, real-time PCR (RT-PCR) analyses were performed on CD11b^+^ cells isolated from the brain of GL261-bearing mice, or from human GBM specimens, and on NCM- and GCM-exposed or cytokine-polarized microglia. A significant higher expression of KCa3.1 (*kcnn4* gene) was observed in CD11b^+^ cells isolated from the ipsilateral cerebral hemisphere of glioma-bearing mice in comparison with the contralateral hemisphere and this difference was totally abolished by TRAM-34 treatment (Figure 3a, left). The expression level of *kcnn4* in CD11b+ cells isolated from the contralateral hemisphere was always comparable with the level measured in control healthy mice (data not shown). In accordance, KCa3.1 level was reduced upon TRAM-34 treatment in M/MΦ cells (Iba1+) infiltrating the tumor area (Figure 3a, center and right). Consistently, when CD11b^+^ cells isolated from human GBM specimens were treated with TRAM-34 for 24 h, a significant reduction of *KCNN4* expression was observed (Figure 3b). Similar results were obtained on cultured microglia upon GCM (Figure 3c) and IL-4 (Figure 3d) treatment, and KCa3.1 inhibition abolished the effect. Taken together, these data suggest that the activity of KCa3.1 channels modulates both microglia phenotype and channel expression.

Modulation of channel expression was confirmed by functional analysis: cytokine-polarized microglia were tested for KCa3.1-mediated currents by patch-clamp recordings. Data shown in Figure 3e demonstrate the presence of large, 5-(4-phenoxybutoxy)psoralen (PAP-1)-sensitive voltage-activated K^+^ channel (Kv1.3) currents for LPS/IFN*γ*-treated microglia, together with barely detectable TRAM-34-sensitive currents (inset, 16.05±7.34 pS/pF). In contrast, IL-4-treated microglia had no significant Kv1.3 currents (not shown), with a more prominent TRAM-34-sensitive component (19.45±12.68 pS/pF) confirming the tendency to a preferential expression of these channels on microglia with anti-inflammatory features.

**Figure 4:**
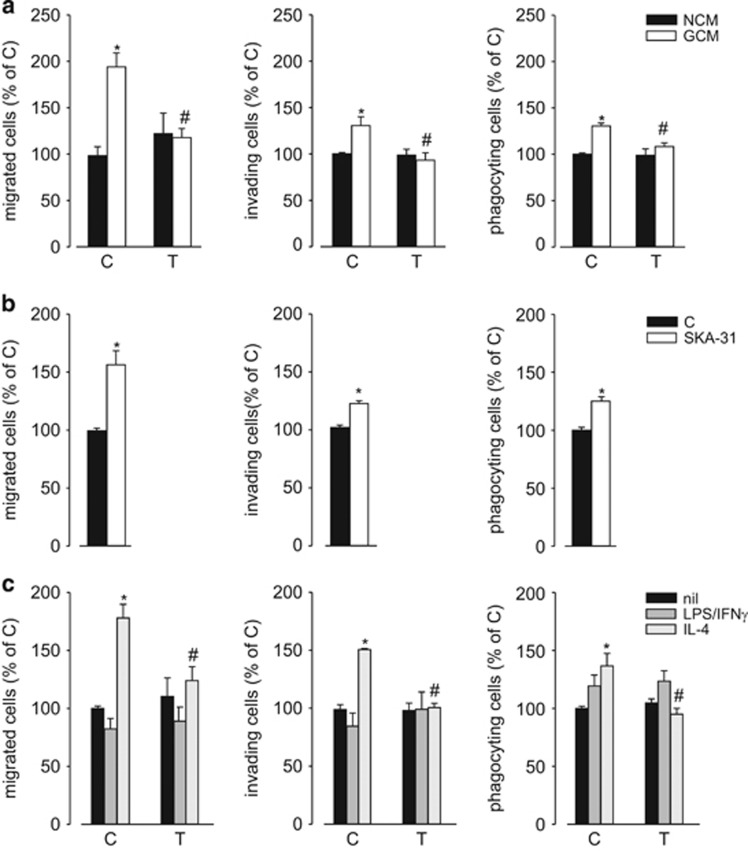
KCa3.1 modulates microglia functional activities. (**a**) NCM- and GCM-treated microglia, in the absence (C) or presence of TRAM-34(T, 2.5 *μ*M) assayed for migration, invasion and phagocytosis. Data are the mean±S.E.M., *P<0.05 *versus* NCM; ^#^*P*<0.05 *versus* GCM/C by two-way ANOVA; *N*=4. (**b**) Untreated (C) or SKA-31 (250 nM) treated microglia assayed for migration, invasion and phagocytosis. Data are the mean±S.E.M., *P<0.05 by Student's *t*-test; *N*=4. (**c**) Untreated, LPS/IFN*γ*- or IL-4-treated microglia in the absence (C) or presence of TRAM-34 (T, 2.5 *μ*M) assayed for migration, invasion and phagocytosis. Data are the mean±S.E.M., *P<0.05 *versus* nil; ^#^*P*<0.05 *versus* LPS/IFN*γ*/C or IL4/C by two-way ANOVA; *N*=4

We have previously shown that GCM exposure modulates microglia migration and phagocytic activity and that KCa3.1 inhibition abolished these effects.^14^ We confirmed these data by performing migration, invasion and phagocytosis assays on primary murine microglia exposed to medium conditioned by GL261 (Figure 4a), U87MG and GL15 (Supplementary Figure S2) glioma cell lines, in the presence of TRAM-34, finding that KCa3.1 block inhibited all the activities potentiated by GCM (Figure 4a). To further support these results, we used a specific activator of KCa3.1, naphtho[1,2-*d*]thiazol-2-ylamine (SKA-31), and found that treatment with this compound increased microglia migration, invasion and phagocytic activity (Figure 4b). Moreover, we analyzed the same activities in primary murine microglia treated with LPS/IFN*γ* or IL-4, demonstrating that only IL-4-treated microglia had increased phagocytic, chemotactic and invasive properties (similarly to GCM-exposed microglia). KCa3.1 inhibition impaired these functional modulations, being ineffective on LPS/IFN*γ*-stimulated microglia (Figure 4c). To further investigate this effect, we decided to study whether cytokine stimulation modulated the expression levels of receptors for chemokines or MMPs. IL-4 treatment induced increased expression of CXCR4, CXCR6 and MMP-9 on microglia, while LPS/IFN*γ* treatment was ineffective (on CXCR6 and MMP-9) or drastically inhibitory (on CXCR4). TRAM-34 abolished the effects of IL-4 on chemokine receptors and MMP-9 expression (Supplementary Figure S3A). Consistently, IL-4-treated cells migrated more toward CXCL12 and CXCL16 (Supplementary Figure S3B). MTT assay excluded "that the effects of GCM and IL-4 was on cell proliferation," some increase being only observed for LPS/IFN*γ*-treated cells (Supplementary Figure S3C).

**Figure 5:**
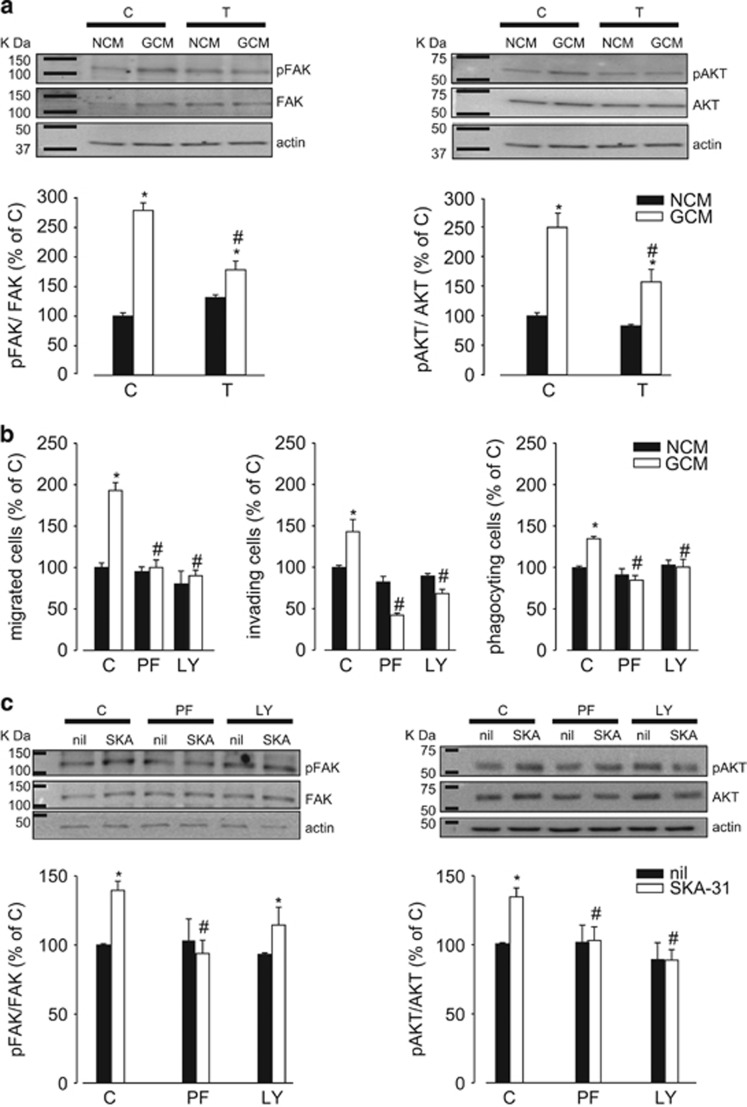
Signaling pathways involved in KCa3.1 channel activation. (**a**) Levels of pFAK/FAK and pAKT/AKT in NCM- and GCM-treated microglia in the absence (C) or presence of TRAM-34 (T, 2.5 *μ*M). Data are the mean±S.E.M., *P<0.05 *versus* NCM; ^#^*P*<0.05 *versus* GCM/C; *N*=5. Representative blots are shown on the top. Actin was used as a loading control, and molecular markers are indicated on the left (KDa). (**b**) Migration, invasion and phagocytosis assays on NCM- and GCM-treated microglia in the absence (C) or presence of PF-228 (PF, 10 *μ*M) or LY294002 (LY, 25 *μ*M). Data are the mean±S.E.M., *P<0,05 *versus* NCM; ^#^*P*<0.05 *versus* GCM/C; *N*=4. (**c**) Levels of pFAK/FAK and pAKT/AKT in untreated or SKA-31-treated (250 nM) microglia in the absence (C) or presence of PF-228 (PF, 10 *μ*M) or LY294002 (LY, 25 *μ*M). Data are shown as mean±S.E.M.; *P<0.05 *versus* NCM; ^#^*P*<0.05 *versus* GCM/C; *N*=8. Top: representative blots are shown on the top. Actin was used as a loading control, and molecular markers are indicated on the left (KDa)

It has been demonstrated that the exposure of microglia to soluble factors released by glioma cells induces the activation of the PI3K/Akt and FAK pathways and that these signaling pathways are involved in cell movement and phagocytosis.^22, 23^ Having demonstrated that anti-inflammatory microglia have increased migratory and phagocytic activity (Figure 4c) and higher functional expression of *kcnn4* (Figure 3), we wanted to verify the hypothesis that KCa3.1 activity could contribute to the phenotype switch of microglia through the modulation of these signaling pathways. We observed that GCM-exposed microglia have increased FAK and AKT phosphorylation, significantly reduced by TRAM-34 treatment, demonstrated both by western blotting and immunofluorescence analysis (Figure 5a and Supplementary Figure S4). Moreover, GCM-induced migration, invasion and phagocytosis were completely inhibited in the presence of FAK (PF-228) and PI3K/AKT (LY294002) inhibitors (Figure 5b). To investigate whether KCa3.1 activation has effect on FAK and PI3K/AKT pathways, cultured microglia were treated with SKA-31, a channel activator. Data shown in Figure 5c demonstrate that both FAK and AKT phosphorylation was higher in SKA-31-treated cells. As FAK activation has been demonstrated to be upstream to the PI3K/AKT pathway in macrophages,^24, 25^ we investigated the cross-talk among these kinases in microglia upon KCa3.1 channel activation. In the presence of PF-228, the effect of SKA-31 on FAK and AKT phosphorylation was inhibited while, in the presence of LY294002, the SKA-31-dependent FAK phosphorylation was maintained, being AKT phosphorylation inhibited (Figure 5c). These data indicate that KCa3.1 activation is upstream to FAK activation and that FAK precedes AKT phosphorylation.

**Figure 6:**
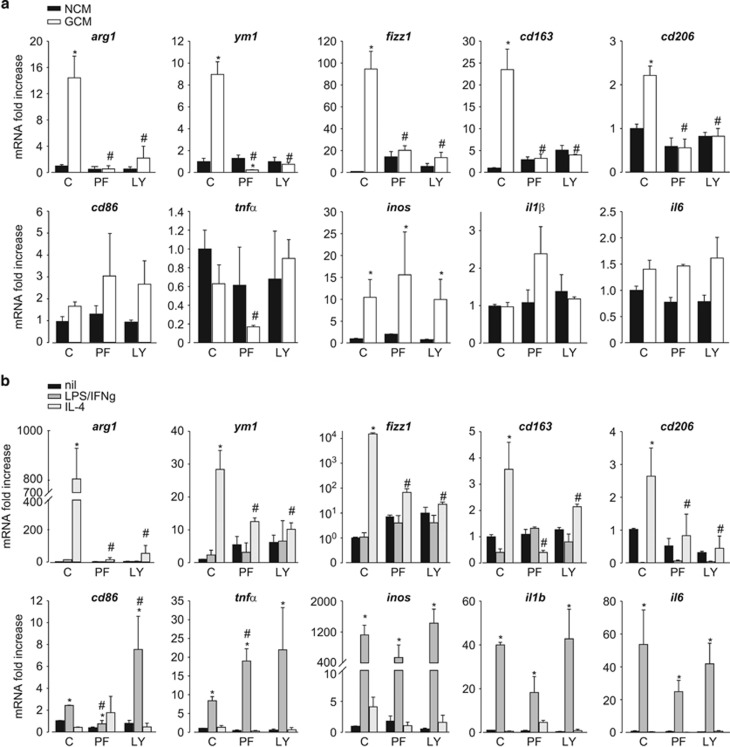
FAK and AKT kinases are required for the anti-inflammatory phenotype of microglia. (**a**) RT-PCR on mRNAs of microglia treated with NCM or GCM in the absence (C) or presence of PF-228 (PF, 10 *μ*M) or LY294002 (LY, 25 *μ*M), assayed for the expression of anti- (*arg1*, *ym1*, *fizz1*, *cd163* and *cd206*) and pro- (*cd86*, *tnfα*, *inos*, *il1β* and *il6*) inflammatory genes. Data are expressed as fold increase and are the mean±S.E.M.; *P<0.05 *versus NCM*; #*P*<0.05 *versus* GCM/C by two-way ANOVA; *N*=8. (**b**) RT-PCR on mRNAs of untreated, LPS/IFN*γ*- or IL-4-treated microglia in the absence (C) or presence of PF-228 (PF, 10 *μ*M) or LY294002 (LY, 25 *μ*M), assayed for the expression of anti- (*arg1*, *ym1*, *fizz1*, *cd163* and *cd206*) and pro- (*cd86*, *tnfα*, *inos*, *il1β* and *il6*) inflammatory genes. Data are expressed as fold increase and are the mean±S.E.M.; *P<0.05 *versus* nil; ^#^*P*<0.05 *versus* LPS/IFN*γ*/C or *versus* IL4/C by two-way ANOVA; *N*=4

To define the role of the FAK/PI3K/AKT pathway in phenotype polarization, microglia were treated with NCM or GCM and LPS/IFN*γ* or IL-4 and incubated with PF-228 or LY294002. Both kinase inhibitors were ineffective on NCM- and LPS/IFN*γ*-treated microglia but prevented GCM- and IL-4-induced expression of anti-inflammatory genes (Figure 6).

## Discussion

GBM is the brain tumor with the lowest survival rate from the time of diagnosis. Its aggressiveness is certainly aided by the ability to produce cytokines and factors involved in tuning down the local immune system response, resulting in an inability to react against cancer cell proliferation and invasion.^26^ One approach to fight GBM is to re-activate the immune system starting with local microglia and infiltrating macrophages, which constitute the first-line innate immune defense in the brain.

In this study, we tested the hypothesis that targeting microglial KCa3.1 channels could represent a therapeutic strategy to counteract GBM malignancy, helping microglia to re-acquire a more pro-inflammatory, antitumor phenotype. This hypothesis is supported by several recent findings demonstrating the ability of the tumor microenvironment to hamper microglia inflammatory reaction, inhibiting the maintenance of the M1-like (pro-inflammatory) phenotype initially induced upon microglia interaction with glioma cells. We demonstrated that the anti-inflammatory M/MΦ phenotype induced by interaction with glioma could be significantly attenuated, in favor of a pro-inflammatory state by modulating the activity of KCa3.1.

Microglia cells isolated from glioma-bearing mice treated with TRAM-34 had a phenotype clearly oriented toward the pro-inflammatory, antitumor state, consistent with the reduction of tumor size observed in these animals. We cannot exclude that, at least *in vivo*, the TRAM-34-induced phenotype switch of M/MΦ could be secondary to a direct effect of the drug on glioma.^14^ However, this would only partially explain the effect, because similar effects on cell phenotype were obtained with CD11b+ cells from human GBM biopsies treated with TRAM-34 and with pure cultured microglia exposed to GCM or IL-4. These data indicate that the block of KCa3.1 is sufficient to hinder microglia phenotype switch, suggesting that these channels could be directly involved in modulating the microglia activation state. This is in line with previous papers demonstrating that KCa3.1 inhibition affects microglia proliferation, p38 MAPK phosphorylation, NF-*κ*B activation and nitric oxide generation.^18, 19^ Similar results were obtained when microglia were stimulated with cytokines: microglia treated with IL-4 displayed increased levels of the anti-inflammatory genes *arg1*, *ym1*, *fizz1*, *cd163* and *cd206*, which were all reduced upon TRAM-34 treatment. Consistently, in IL-4-treated cells, we observed higher levels of *kcnn4* and recorded TRAM-34-sensitive currents.

Taken together, these data support the hypothesis that KCa3.1 is involved in determining microglia phenotype, in particular shifting glioma-induced cell polarization toward a pro-inflammatory state. Interestingly, a shift of IL-4-polarized macrophage toward a pro-inflammatory phenotype was also described for another cation channel, TRPM7,^27^ and a recent reports describe that CSFR-1 inhibition has similar effects on the polarization of tumor-associated macrophages (TAM), downregulating M2-related genes, with consequent antitumor effects.^28^ In accordance, inhibition of the mTOR kinase has been reported to induce the polarization of glioma-induced microglia toward the pro-inflammatory state.^29^ It is known that glioma-exposed microglia have an increased tendency to migrate, invade and phagocyte;^14^ here we also observed that KCa3.1 inhibition affected these microglia functions, likely contributing to the antitumor activity of TRAM-34, in addition to the direct effect on glioma.^14^ We also reported that microglia treated with the KCa3.1 channel activator SKA-31, or polarized by IL-4, had similar increased migratory, invasive and phagocytic activities, all significantly reduced by KCa3.1 inhibition. Microglia migration varies according to their activation state.^30, 31^ We report that IL-4-treated microglia upregulated the chemokine receptors CXCR4 and CXCR6 and the MMP-9, all effects being abolished by TRAM-34. Interestingly, LPS/IFN*γ* treatment did not modify CXCR6 or MMP-9 expression, strongly inhibiting CXCR4 expression. This reduction could contribute to the antitumor effect reported for microglia in the pro-inflammatory state.^12^ IL-4 treatment consistently increased microglia migration toward CXCL12 and CXCL16. Interestingly, similar reduction of MMP-12 is induced by TRAM-34 treatment of CD11b+ cells isolated from GBM patients. These results confirm that the anti-inflammatory phenotype of microglia can be reverted by TRAM-34, which, however, also affects some classic pro-inflammatory features of microglia.^16, 18, 19^

Remarkably, we report that TRAM-34 did not affect microglia functions (migration, invasion, phagocytosis) in the absence of stimulation or upon LPS/IFN*γ* treatment, in contrast to the inhibitory effects observed on activating pathways induced by inflammatory stimuli, such as oligomeric amyloid and LPS.^19^ This could be explained by the lower expression level of *kcnn4* and the lower functional expression of KCa3.1 in comparison with anti-inflammatory phenotype, in our experimental systems, that comprise *in vivo*, *ex vivo* and *in vitro* conditions.

To describe the intracellular pathways involved in KCa3.1-mediated control of microglia phenotype, we analyzed FAK and PI3K/AKT signaling that have been demonstrated to be activated in TAMs and involved in the M2-like microglia polarization.^23^ We demonstrated that both kinases are essential in movement, phagocytosis and invasion of GCM- and IL-4-treated cells; that FAK and PI3K/AKT signaling in microglia is downstream of KCa3.1 activation and that the block of FAK phosphorylation hampers SKA-31-induced PI3K/AKT activation. We hypothesize that KCa3.1 activation could induce local Ca^2+^ influx leading to FAK and AKT phosphorylation^32, 33^ and that these signals are involved in GCM- and IL-4-mediated microglia activation and polarization. KCa3.1 inhibition with TRAM-34 inhibited all these events, promoting the induction of antitumor signals.

Our data suggest that, in addition to being involved in modulating microglia phenotype, KCa3.1 channels are differentially expressed in the different activation states of microglia. Microglia isolated from the brain of glioma-bearing mice and from biopsies of patients with glioma had significant upregulation of *kcnn4*, as well as microglia exposed to GCM or treated with IL-4 (see also Ferreira *et al.*^34^). In all these samples, KCa3.1 inhibition reduced *kcnn4* expression, as already described in human lung myofibroblasts and vascular smooth muscle cells,^35, 36, 37^ suggesting a clear relation between microglia activation state and the control of KCa3.1 transcription. A relation between KCa3.1 expression level and activation was already reported in T cells, where a Ca^2+^-dependent mechanism involves JNK and AP1 (c-Fos/c-Jun heterodimer) complex binding to the *kcnn4* promoter.^38^

In summary, based on the data herein presented, we propose KCa3.1 as a possible marker of M/MΦ in the pro-tumor state, and thus a potential therapeutic target to induce GBM-associated M/MΦ to re-acquire a pro-inflammatory, antitumor activity. Of note, and important for future therapeutic use, one TRAM-34 analog, Senicapoc (ICA-17043), has been already used in phase-3 clinical trials, where it was considered safe, with no clinically relevant changes in vital signs and physical examination.^39^

## Materials and Methods

### Animals and cell lines

Experiments were approved by the Italian Ministry of Health in accordance with the ethical guidelines on use of animals from the EC Council Directive 2010/63/EU. We used C57BL/6 mice (Charles River, Calco, Italy). Human GBM (GL-15 and U87MG) and murine glioma (GL261) cell lines were cultured in FBS-supplemented DMEM.

### Microglia polarization

Microglia were obtained from cerebral cortices of postnatal day 0–2 mice^40^ and treated for 24–48 h with LPS 100 ng/ml+IFN*γ* 20 ng/ml (ImmunoTools, Friesoythe, Germany) or IL-4 20 ng/ml (ImmunoTools) for cell polarization.

### Patch clamp

Patch-clamp recordings were obtained using glass electrodes (2–4 MΩ) filled with intracellular solution (see composition in Supplementary Methods). Currents were recorded by whole-cell configuration at RT with EPC-10HEKA amplifier (HEKA, Lambrecht/Pfalz, Germany). K^+^ currents were elicited with voltage ramps (−120 mV to +40 mV, 200 ms) every 10 s. KCa3.1 conductances were calculated from the slope of the TRAM-34-sensitive KCa current between −80 and −75 mV, where KCa3.1 currents are not 'contaminated' by Kv1.3 (activable at voltages >−40 mV) or inward rectifier K^+^ currents (appreciable at voltages <−80 mV). Cell capacitance was continuously monitored. KCa3.1 current density was determined as the TRAM-34-sensitive slope conductance/cell capacitance. The prominent Kv current in pro-inflammatory microglia was blocked with Kv1.3 blocker PAP-1 (1 *μ*M).

### Chemotaxis and invasion assays

Microglia stimulated with NCM or GCM, and cytokine-polarized microglia were treated for 24 h with TRAM-34 (2.5 *μ*M) or without. Cells were incubated 3 h at 37 °C with 10% FBS in the lower chamber as chemoattractant or with chemokines (CXCL12, 50 nM; CXCL16, 1 nM, Peprotech, London, UK). For invasion assay, microglia were plated on matrigel-coated transwells (BD-Falcon, Milan, Italy) and the movement toward GCM (from GL261, GL15 or U87MG) was investigated in the presence of TRAM-34 (2.5 *μ*M) for 24 h.

### Phagocytosis

Microglial cells were stimulated with GCM for 24 h with TRAM-34 (2.5 *μ*M). Alternatively, polarized microglia were treated with TRAM-34 (2.5 *μ*M) for 24 h. Red fluorescent FluoSpheres (0.03%) (Invitrogen, Monza, Italy) were added for 1 h, and the number of spheres per cell was counted.^14^

### Real-time PCR

GCM-stimulated or cytokine-polarized microglia were treated with TRAM-34 (2.5 *μ*M). After 24 h, total RNA was extracted with Trizol reagent (Invitrogen), quantified and retro-transcripted using IScript Reverse Transcription Supermix (Bio-Rad, Milan, Italy). RT-PCR was carried out in a I-Cycler IQ Multicolor RT-PCR Detection System (Bio-Rad) using SsoFast Eva Green Supermix (Bio-Rad). The PCR protocol consisted of 40 cycles of denaturation at 95 °C for 30 s and annealing/extension at 58 °C for 30 s. The Ct values from each gene were normalized to the Ct value of GAPDH. Relative quantification was performed using the 2^−ΔΔCt^ method and expressed as fold increase. Primer sequences are in Supplementary Table S2.

### Form factor calculation

Microglia were seeded on glass coverslips, treated as necessary, fixed, permeabilized, blocked and stained with Alexa-Fluor 488 Phalloidin (Invitrogen) for 20 min together with Hoechst. Fluorescent images were processed using the MetaMorph 7.6.5.0 software (Molecular Device, Sunnyvale, CA, USA), and form factor was calculated according the formula: 4*π* area/perimeter^2^.^41^

### Western blotting analysis

For protein phosphorylation analysis, protein samples were separated on 8.75% SDS-polyacrylamide gel electrophoresis and analyzed by western immunoblot using the following primary antibodies: pFAK (Tyr397, Santa Cruz Biotechnology, Santa Cruz, CA, USA) 1 : 200, pAKT (Ser473, Cell Signaling, Danvers, MA, USA) 1 : 1000, FAK (Santa Cruz Biotechnology) 1 : 200, AKT (Cell Signaling) 1 : 1000, and Actin (Sigma Aldrich, Milan, Italy) 1 : 2000; HRP-tagged goat anti-rabbit IgG was used as a secondary antibody (1 : 2000; Dako, Cernusco sul Naviglio, Milan, Italy); the detection was performed through the chemiluminescence assay Immun-Star Western C Kit (Bio-Rad). Densitometric analysis was carried out with the Quantity One software (Bio-Rad).

### Tumor cell implantation and mice treatment

Eight-week-old male C57BL/6 mice were anesthetized with chloralhydrate (400 mg/kg, i.p.) and stereotaxically injected with 1 × 10^5^ GL261 cells in 5 *μ*l PBS, 2 mm right and 1 mm anterior to the bregma in the striatum at 3 mm depth with a Hamylton syringe (Bonaduz, Switzerland). After 7 days, mice were daily treated with TRAM-34 (120 mg/Kg i.p.) or vehicle (peanut oil).

### Tumor volume analysis

After 17 days from GL261 injection, animals were killed and the brains were isolated. Tumor volume was evaluated with hematoxylin–eosin staining as previously described.^42^ Briefly, after staining, brain slices (20 *μ*m of thickness) were analyzed by the Image Tool 3.0 software (University of Texas, Health Science Center, San Antonio, TX, USA). to measure the tumor area, and volume was calculated according to the formula (volume=*t* × Σ*A*), where *A*=tumor area/slice and *t*=thickness.^43, 44^

### Isolation of CD11b^+^ cells

After 17 days from GL261 injection, mice were deeply anesthetized and perfused with ice-cold PBS. Brains were removed and digested with trypsin (0.25 mg/ml). Tissue suspension was applied to a 30-*μ*m cell strainer, labeled with CD11b MicroBeads and isolated accordingly to the manufacturer's instructions (Miltenyi Biotec, Calderara di Reno, Bologna, Italy). CD11b+ cell purity (99%) was verified as reported.^42^

### Immunofluorescence

Coronal brain sections (20 *μ*m) were washed in PBS, blocked (3% goat serum in 0.3% Triton X-100) for 1 h at RT and incubated overnight at 4 °C with specific antibodies, Iba1 (Dako) 1 : 500 and KCa3.1 (Alomone, Jerusalem, Israel) 1 : 100. Brain slices were stained with the fluorophore-conjugated secondary antibodies and Hoechst for nuclei visualization and analyzed using a fluorescence microscope. Signals co-localization was analyzed measuring the average fluorescence intensity (pixel) of merged signals.

### Isolation of CD11b^+^cells from human GBM

Tumor specimens obtained from adult patients who gave informed consent to the research proposals (Neurosurgery Departments, Neuromed and Policlinico Umberto I) were processed as described above to isolate CD11b^+^ cells. See Supplementary Table S1 for patients' details. Cells were treated with TRAM-34 (2.5 *μ*M) for 24 h and mRNAs were analyzed by RT-PCR for human pro- and anti-inflammatory markers (primer sequences are listed in Supplementary Table S2).

### Statistical analysis

Data are expressed as the means±S.E.M. Student's *t*-test, paired *t*-test, one-way or two-way analysis of variance (ANOVA) was performed. A value of *P*<0.05 was considered significant. All statistical analyses were carried out using the Sigma Plot 11.0 Software (Systat Software GmbH, Erkrath, Germany).

## Abbreviations

M/MΦ: microglia/macrophages
KCa3.1: Ca^2+^activated K^+^channel
GBM: glioblastoma multiforme
TRAM-34: 1-[(2-Chlorophenyl)diphenylmethyl]-1*H*-pyrazole
IL: interleukin
LPS: lipopolysaccharide
IFN: interferon
FAK: focal adhesion kinase
PI3K: phosphoinositide-3 kinase
Akt: protein kinase B
GCM: glioma-conditioned medium
NCM: non-conditioned medium
RT-PCR: real-time PCR
PAP-1: 5-(4-phenoxybutoxy)psoralen
Kv1.3: voltage-activated K^+^ channel
SKA-31: naphtho[1,2-*d*]thiazol-2-ylamine
