# Supplementary material for: KCa3.1 inhibition switches the phenotype of glioma-infiltrating microglia/macrophages
Source: Cell Death Dis. 2016 Apr 7;7(4):e2174–. doi: 10.1038/cddis.2016.73 (PMC4855657; doi:10.1038/cddis.2016.73)
Supplement: Supplementary Information [file cddis201673x1.doc]

**SUPPLEMENTARY METHODS**

**MTT Assay**. To evaluate microglia viability, cells were exposed to specific treatments and then incubated with 0.5 mg/mL MTT in growing medium for 2 h at 37 °C; medium was then aspired, cells were treated with DMSO and incubated at 37 °C for 10 min. Samples were analyzed with a microplate reader at 490 nm (and at 630 nm to subtract the background).

**Immunofluorescence**. Microglia were plated on coverslips, treated with NCM or GCM in presence or absence of TRAM-34 (2.5 μM), washed in PBS, fixed in 4% PFA, permeabilized (Triton X-100 0.1%) and blocked (1% BSA) for 1 h at RT, and incubated overnight at 4 °C with pFAK (Tyr397, Santa Cruz Biotechnology, USA) 1:100 or pAKT (Ser473, Cell Signaling, USA) 1:100. Cells were stained with the fluorophore-conjugated secondary antibodies and Hoechst for nuclei visualization and analyzed using a fluorescence microscope. pFAK and pAKT levels were analyzed measuring the mean fluorescence intensity of each signal.

**SUPPLEMENTARY FIGURE LEGENDS**

**Supplementary Figure 1**. KCa3.1 inhibition reverts cytokine-induced microglia polarization.(**A**) RT-PCR on mRNAs of untreated, LPS/IFNγ or IL-4 treated microglia in the absence (C) or presence of TRAM-34 (T, 2.5μM), assayed for the expression of anti-inflammatory (*arg1, ym1, fizz1, cd163 and cd206*) and pro-inflammatory (*cd86, tnfα, inos, il1β and il6*) genes. Data are expressed as fold increase and are the mean ± SEM; *p<0.05*vs*nil*;* #p<0.05 *vs* LPS/IFNγ/C or *vs*IL4/C by two-way ANOVA; *N*=4. (**B**) Untreated, LPS/IFNγ or IL-4 treated microglia in the absence (C) or presence of TRAM-34 (T, 2.5μM), stained with phalloidin (green) and Hoechst (blue). Form factor values are shown in the graph and are the mean ± SEM *p<0.05 *vs* nil*;* #p<0.05 *vs* LPS/IFNγ/C or *vs* IL4/C by two-way ANOVA; *N*=4.

**Supplementary Figure 2**. KCa3.1 modulates GCM-induced functional activities of microglia. Microglia exposed to NCM, GL15- or U87MG-conditioned medium (CM) in the absence(C) or presence of TRAM-34 (2.5μM) were assayed for migration (**A**), invasion (**B**) and phagocytosis (**C**). Data are the mean ± SEM, *p<0.05*vs* NCM; # p<0.05 vs GCM;*N*=4.

**Supplementary Figure 3**. Effect of microglia polarization on cell functions.(**A**) RT-PCR on mRNAs of untreated, LPS/IFNγ or IL-4 treated microglia in the absence (C) or presence of TRAM-34 (T, 2.5 μM), analyzed for the expression of *cxcr4*, *cxcr6* and *mmp9* genes. (**B**) Untreated, LPS/IFNγ or IL-4 treated microglia were assayed for chemotaxis towards CXCL12 or CXCL16. Data are the mean ± SEM, *p<0.05 *vs* C; #p<0.05 *vs* nil by two-way ANOVA; *N*=3. (**C**) Proliferation index (evaluated by MTT assay) performed on microglia (left) treated for 24 and 48 h with NCM or GCM or (right) treated for 48 h with LPS/IFNγ or IL-4. Data are expressed as % *vs* NCM (left) or untreated cells (right) and are the mean ± SEM; *p<0.05 *vs* nil; *N*=4.

**Supplementary Figure 4**. Signaling pathways involved in KCa3.1 channel activation. (**A**) Representative full blot of pFAK, FAK, pAKT and AKT in NCM and GCM-treated microglia in the absence (C) or presence of TRAM-34 (T, 2.5 µM). Molecular markers are indicated on the left (KDa). (**B)** NCM and GCM-treated microglia in the absence (C) or presence of TRAM-34 (T, 2.5 µM) were assayed for pFAK and pAKT by immunofluorescence. Data are the mean fluorescence intensity ± SEM, *p<0.05 *vs* NCM; #p<0.05 *vs* GCM/C; *N*=3. Representative pictures are shown on the top (pFAK and pAKT in red, Hoechst in blue, scale bar = 20μm).

**Supplementary Table 1**. Data from GBM patients.

**Supplementary Table 2**. List of primers used for Real-Time PCR experiments.
