# Supplementary material for: KCa3.1 inhibition switches the phenotype of glioma-infiltrating microglia/macrophages
Source: Cell Death Dis. 2016 Apr 7;7(4):e2174–. doi: 10.1038/cddis.2016.73 (PMC4855657; doi:10.1038/cddis.2016.73)
Supplement: Supplementary Table 1 [file cddis201673x2.doc]

Supplementary Table 1. GBM patient information

| **GBM :ID** | **Gender** | **Age** | **GBM grade (WHO)** | **Relapse** |
| --- | --- | --- | --- | --- |
| 01 | M | 62 | IV | No |
| 02 | F | 60 | IV | Yes |
| 03 | F | 36 | IV | Yes |
| 04 | M | 64 | IV | No |

Grimaldi et al., Suppl. Table 1
