# Supplementary material for: KCa3.1 inhibition switches the phenotype of glioma-infiltrating microglia/macrophages
Source: Cell Death Dis. 2016 Apr 7;7(4):e2174–. doi: 10.1038/cddis.2016.73 (PMC4855657; doi:10.1038/cddis.2016.73)
Supplement: Supplementary Table 2 [file cddis201673x3.doc]

**Supplementary Table 2. Primers used for Real Time-PCR analysis.**

| **Gene** | **Species** | **Primer Forward (5’-3’)** | **Primer Reverse (5’-3’)** |
| --- | --- | --- | --- |
| *arg1*  *cd86*  *CD163*  *cd206*  *CXCL10*  *cxcr4*  *cxcr6*  *fizz1*  *GAPDH*  ***g****apdh*  *il1b*  *il6*  *IL12A*  *inos*  *kcnn4*  *KCNN4*  *mmp9*  *MMP12*  *NOS2*  *TGFB1*  *TNF*  *tnfa*  *ym1* | mouse  mouse  human  mouse  human  mouse  mouse  mouse  human  mouse  mouse  mouse  human  mouse  mouse  human  mouse  human  human  human  human  mouse  mouse | CTCCAAGCCAAAGTCCTTAGAG  AGAACTTACGGAAGCACCCA  TCTGGCTTGACAGCGTTTC  CAAGGAAGGTTGGCATTTGT  GTGGCATTCAAGGAGTACCTC  CCATGGCTGACTGGTACTTT  CCCTTTTGGGCCTATGCAG  CCAATCCAGCTAACTATCCCTCC  CCCCTTCATTGACCTCAACTAC  TCGTCCCGTAGACAAAATGG  GCAACTGTTCCTGAACTCAACT  GATGGATGCTACCAAACTGGA  CTCCTGGACCACCTCAGTTTG  ACATCGACCCGTCCACAGTAT  GGCTGAAACACCGGAAGCTC  GGCTGAAACACCGGAAGCTC  TAGCTACCTCGAGGGCTTCC  AGGAATCGGGCCTAAAATTG  CAGCGGGATGACTTTCCAA  CCAACTATTGCTTCAGCTCCAC  CCCAGGGACCTCTCTCTAATCA  GTGGAACTGGCAGAAGAG  CAGGTCTGGCAATTCTTCTGAA | AGGAGCTGTCATTAGGGACATC  GGCAGATATGCAGTCCCATT  TGTGTTTGTTGCCTGGATT  CCTTTCAGTCCTTTGCAAGT  TGATGGCCTTCGATTCTGGATT  TCAGGAGGAGGGCTGGGATC  ATGCCTCGAAGAGTTTTGCAC  ACCCAGTAGCAGTCATCCCA  GATGACAAGCTTCCCGTTCTC  TTGAGGTCAATGAAGGGGTC  ATCTTTTGGGGTCCGTCAACT  TCTGAAGGACTCTGGCTTTG  GGTGAAGGCATGGGAACATT  CAGAGGGGTAGGCTTGTCTC  CAGCTCTGTCAGGGCATCCA  CAGCTCTGTCAGGGCATCCA  GTGGGACACATAGTGGGAGG  TGCTTTTCAGTGTTTTGGTGA  AGGCAAGATTTGGACCTGCA  GTGTCCAGGCTCCAAATGTAGG  AGCTGCCCCTCAGCTTGAG  CCATAGAACTGATGAGAGG  GTCTTGCTCATGTGTGTAAGTGA |

Grimaldi et al., Suppl Table 2
