# Supplementary figures and images for: KCa3.1 inhibition switches the phenotype of glioma-infiltrating microglia/macrophages
Source: Cell Death Dis. 2016 Apr 7;7(4):e2174–. doi: 10.1038/cddis.2016.73 (PMC4855657; doi:10.1038/cddis.2016.73)

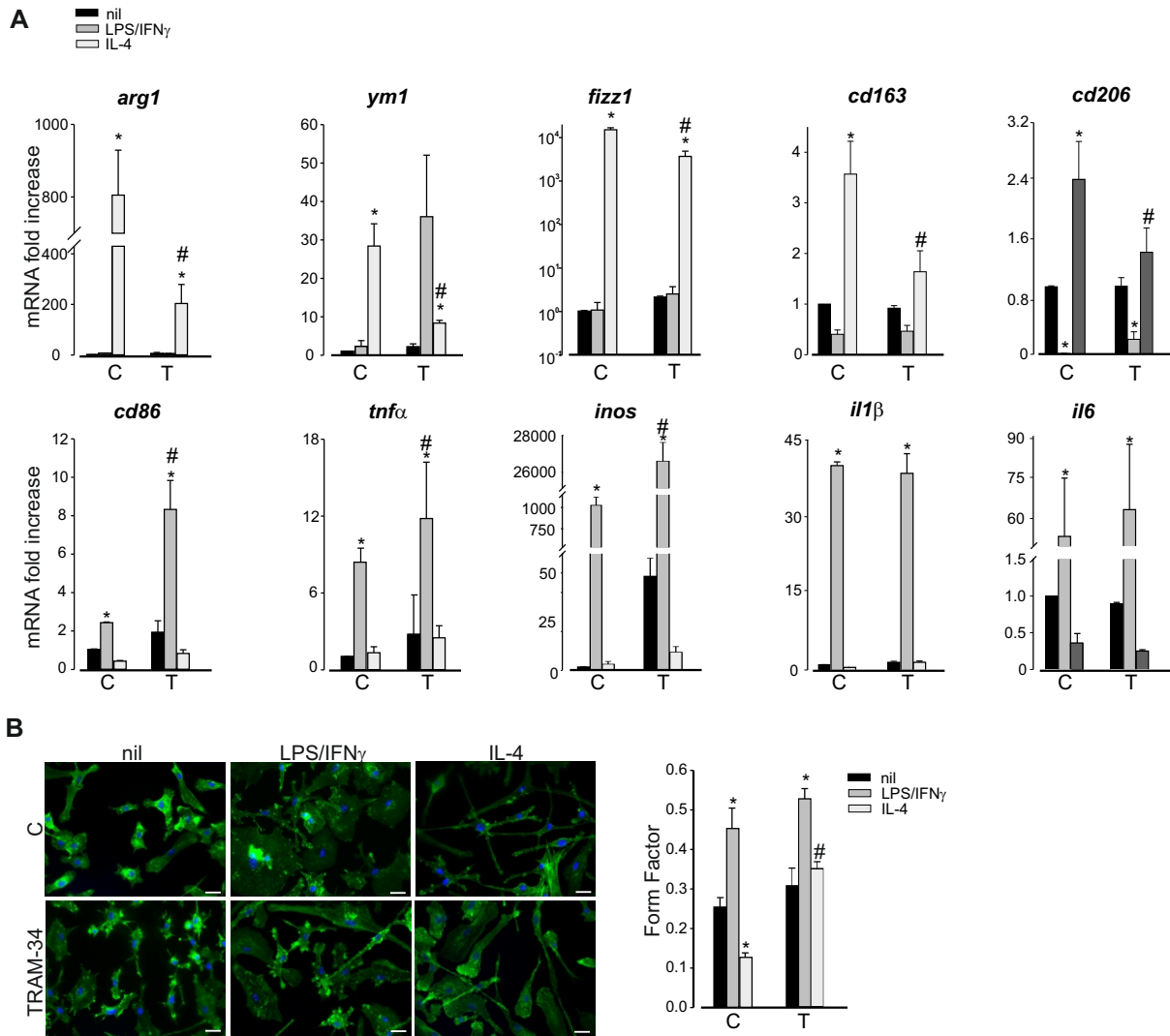

Supplement: Supplementary Figure 1 [file cddis201673x4.pdf]

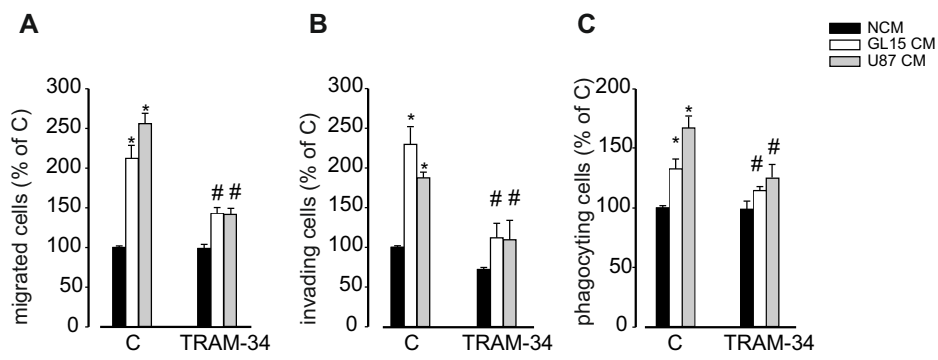

Supplement: Supplementary Figure 2 [file cddis201673x5.pdf]

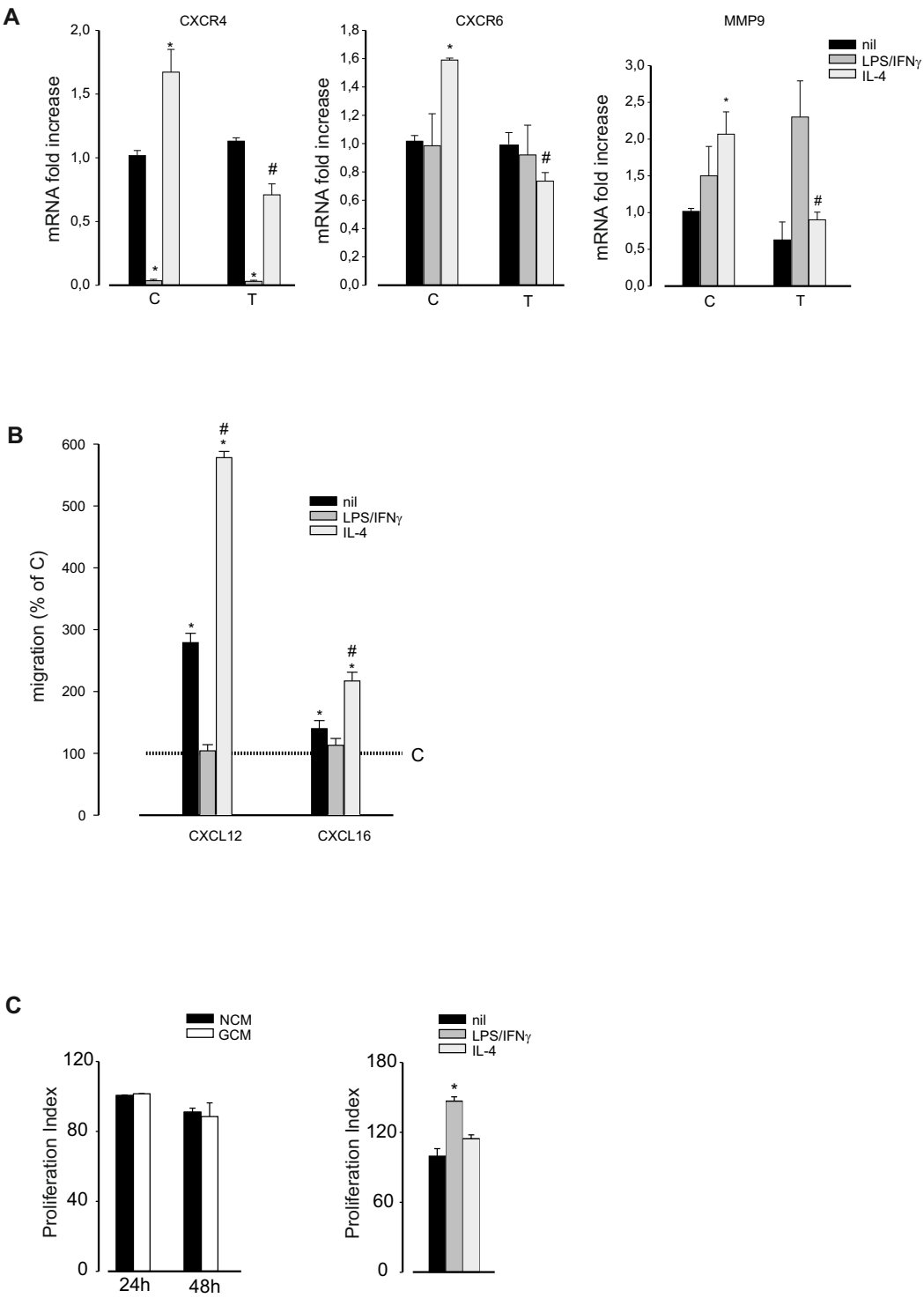

Supplement: Supplementary Figure 3 [file cddis201673x6.pdf]

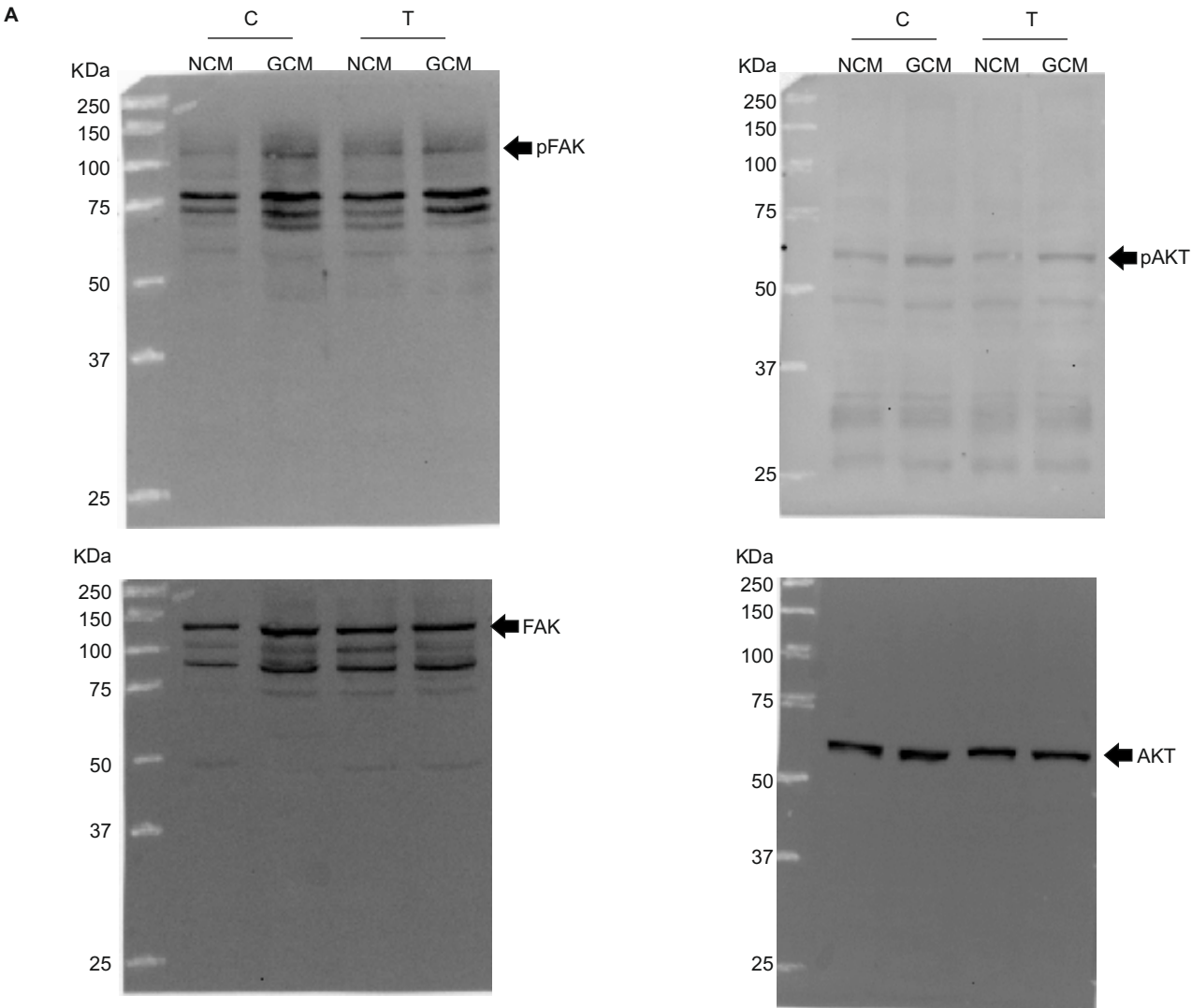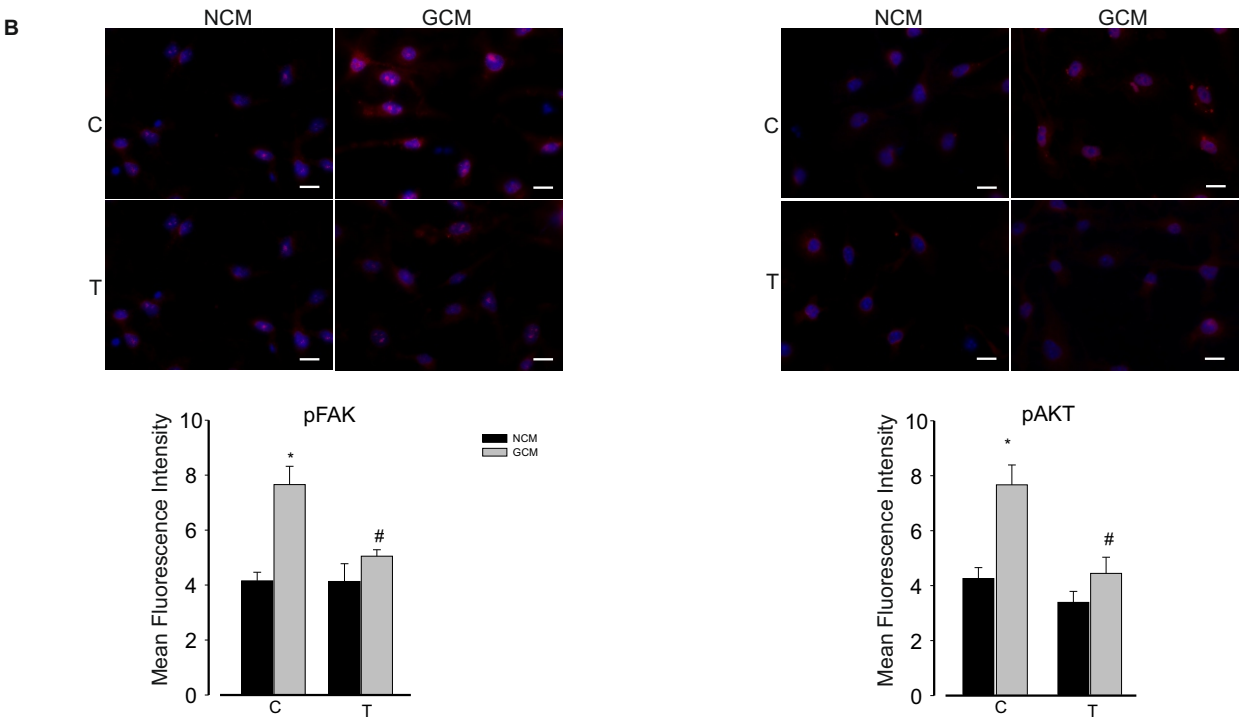

Supplement: Supplementary Figure 4 [file cddis201673x7.pdf]
